# Association between prior percutaneous epidural neuroplasty and distal postoperative spinal epidural hematoma after lumbar surgery: case series and exploratory analysis

**DOI:** 10.3389/fsurg.2026.1863930

**Published:** 2026-09-24

**Authors:** Tae Hoon Yang, In-Suk Bae, Hee In Kang, Jae Hoon Kim, Cheolsu Jwa

**Affiliations:** Department of Neurosurgery, Nowon Eulji Medical Center, Seoul, Republic of Korea

**Keywords:** decompression, epidural hematoma, fusion, lumbar spinal stenosis, percutaneous epidural neuroplasty

## Abstract

**Background:**

Postoperative spinal epidural hematoma (SEH) is a rare but serious complication of spinal surgery. This study explored whether prior percutaneous epidural neuroplasty (PEN) was associated with symptomatic postoperative SEH occurring at a distal level, defined as completely caudal to the surgical decompression site after lumbar spinal surgery.

**Methods:**

We retrospectively reviewed 232 consecutive patients who underwent 1- or 2-level lumbar spinal surgery at a single institution between 2020 and 2024. Clinical, surgical, and historical factors were compared between patients who developed symptomatic postoperative SEH requiring revision surgery and those who did not. Due to the low event rate, statistical analysis was restricted to descriptive metrics and Fisher's exact test to explore the association between prior PEN and distal SEH development.

**Results:**

A total of 232 consecutive patients who underwent lumbar spinal surgery were included. Four patients (1.72%) developed postoperative SEH requiring reoperation. Notably, three of these four cases (75.0%) developed hematomas at a distal level entirely caudal to the surgical site. In the primary distal SEH analysis, all three distal cases had a history of prior PEN, compared to 16.2% (37/229) of patients who did not develop a distal SEH (*P* = 0.005). Because one cell in the 2 × 2 table was zero, the exact odds ratio was unestimable. In an exploratory univariate analysis using the broader SEH endpoint, prior PEN was more frequent in the SEH group than in the non-SEH group (75.0% vs. 16.2%; Fisher's exact test *P* = 0.017; exact odds ratio [OR], 15.49; 95% confidence interval [CI], 1.57–152.99).

**Conclusions:**

In this small series, prior PEN demonstrated an exploratory association with symptomatic, distal level postoperative SEH. Given the small number of events and wide confidence intervals, this finding should be interpreted strictly as hypothesis-generating. In symptomatic postoperative patients with a history of caudal epidural interventions, clinicians should maintain awareness and consider extending MRI coverage caudally beyond the index operated level.

## 1 Introduction

Although rare, postoperative spinal epidural hematomas (SEHs) are a well-recognized complication of spinal surgery (1). SEHs typically arise from the disruption of the low-pressure, valveless epidural venous plexus and should be suspected when a patient develops new, progressive neurological deficits or severe atypical pain after surgery (2, 3). However, postoperative imaging frequently reveals small, asymptomatic fluid or blood collections at the operative site even in the absence of clinically significant mass effect, which can complicate clinical interpretation (1, 4).

Previous literature has identified several risk factors for surgical-site SEH, including advanced age, multilevel surgery, preoperative coagulopathy, anticoagulant or antiplatelet therapy, substantial intraoperative blood loss, postoperative hypertension, and early mobilization (5, 6). Prior revision surgery at the same operative level has also been reported as a potential risk factor because of technical difficulty and scar-related bleeding (4). However, these studies describe hematomas expanding directly at the index surgical site, and reported associations vary across studies due to differences in perioperative practices and the overall rarity of the condition.

To date, no previous study has focused specifically on postoperative SEHs occurring exclusively at a distal level (completely caudal to the decompressed segments) or on their potential association with prior interventional procedures like percutaneous epidural neuroplasty (PEN). Distal SEHs present a unique diagnostic challenge: routine postoperative imaging often targets only the index surgical levels, potentially missing an ongoing caudal mass effect and delaying necessary treatment.

Therefore, the purpose of this study was to present a descriptive case series of distal postoperative SEH with neurological deterioration and to provide an exploratory, hypothesis-generating evaluation of the potential association between a history of PEN and distal level SEH within a single-center cohort.

## 2 Methods

### 2.1 Ethics

This study was approved by the Institutional Review Board of the authors' institution and was conducted in accordance with the Declaration of Helsinki (IRB No. 2025-08-029). The requirement for informed consent was waived due to the retrospective and anonymized nature of the chart review. All patient data were handled in accordance with the tenets of the Declaration of Helsinki.

### 2.2 Study population

We retrospectively reviewed the medical records of patients with lumbar degenerative disease who underwent lumbar spinal surgery between January 1, 2020, and December 31, 2024. Eligible patients had degenerative lumbar spinal stenosis and underwent 1- or 2-level lumbar spinal decompression, with or without instrumented fusion, for intractable back pain, leg pain, or neurogenic claudication.

Patients were excluded if they had structural, pathologic, or traumatic vertebral fractures, active spinal infection, a history of prior revision surgery at the same index operative level, severe cardiopulmonary disease, cognitive impairment, neurological disease preventing reliable communication, or incomplete medical records.

Clinically significant distal level postoperative SEH was defined as any postoperative hematoma at a distal level that necessitated urgent surgical evacuation. Distal level was strictly defined *a priori* as a postoperative epidural hematoma whose maximal compressive component was located entirely caudal, at least one vertebral level below the lowest instrumented or decompressed surgical segment. In multilevel procedures, the most caudal operated level was used as the reference point. Mixed-level hematomas involving both operative and distal components were classified according to the level of maximal compression.

### 2.3 Data collection

All data were obtained from official patient medical records. Demographic data (age, sex, weight, height, and calculated body mass index (BMI), and comorbidities such as hypertension, diabetes mellitus, liver disease, and prior stroke history, were collected together with anticoagulant medication status. Preoperative laboratory parameters included hemoglobin, hematocrit, platelet count, prothrombin time (PT), activated partial thromboplastin time (aPTT), and the international normalized ratio (INR).

Perioperative data collected included the surgery type, surgical level, number of exposed levels, operative time, estimated blood loss (EBL), drain use, drain removal timing, topical hemostatic agent use, intraoperative dural tear or cerebrospinal fluid leak, and venous thromboembolism (VTE) prophylaxis. Body mass index (BMI) was calculated as weight (kg) divided by height squared (m^2^).

Prior PEN exposure was defined as a documented history of undergoing a caudal-approach catheterization for epidural adhesiolysis within the affected lumbar region prior to the index surgery. Because these details were not uniformly documented in the historical records, complete procedural characterization (e.g., target level, type of injectate, volume of injectate) was not possible for all patients across the broader cohort.

### 2.4 Statistical analysis

Continuous variables are presented as mean ± standard deviation. Categorical variables are presented as counts and percentages using explicit denominators. Continuous variables were compared using the Mann–Whitney *U*-test. Categorical variables were analyzed using Fisher's exact test due to the low event rate. Exact odds ratios (ORs) and 95% confidence intervals (CIs) were computed, with statistical significance set at *P* < 0.05.

Because only four outcome events occurred out of 232 patients, formal multivariable logistic regression was omitted because of model instability, overfitting risk, and limited interpretability. For the primary distal postoperative SEH analysis, one cell in the 2 × 2 contingency table was zero; therefore, the exact conditional odds ratio was not estimable in the usual manner and approached infinity. Consequently, we report the exact Fisher's exact test *p*-value together with the lower bound of the exact 95% CI, with an explanatory footnote in the table. Given these constraints, this analysis should be regarded strictly as exploratory and hypothesis-generating, and residual confounding cannot be excluded. All calculations were performed using R software, version 4.2.2 (R Foundation for Statistical Computing, Vienna, Austria).

## 3 Results

### 3.1 Demographic characteristics of the patients

A total of 232 consecutive patients met the inclusion criteria during the study period. Of these, 154 patients (66.4%) underwent decompression-only procedures (including discectomy or laminectomy) and 78 patients (33.6%) underwent instrumented fusion surgery. The most frequently operated level was L4–5, followed by L5–S1 and L3–4.

All patients had a surgical drain placed and received uniform mechanical VTE prophylaxis via anti-embolism stockings after surgery; chemoprophylaxis for VTE was not utilized in this cohort. Drains were removed when output fell below 20 mL over 24 h. In patients receiving anticoagulants, medications were withheld for 5 days before surgery and resumed after drain removal. Group-level distributions for anticoagulant use are tabulated, though specific agent types and exact post-surgical resumption days were not uniformly recoverable from historical files.

Four patients (1.72%) developed new, progressive postoperative neurological deficits or severe atypical pain requiring revision surgery and were diagnosed with postoperative SEH. Notably, three of these four cases (75.0%) demonstrated hematomas located at a distal level entirely caudal to the surgical margins.

### 3.2 Primary outcome: distal SEH cases

Using distal postoperative SEH as the primary endpoint, all three distal SEH cases (100.0%) had a history of prior PEN, compared to only 16.2% (37/229) of patients in the non-distal SEH cohort (*P* = 0.005). Because of the zero-cell structure in the 2 × 2 contingency table, the exact odds ratio approached infinity and was conventionally unestimable, yielding a lower bound of the exact 95% CI of 1.528.

Baseline demographic and clinical characteristics stratified by the presence of distal postoperative SEH are summarized in Table 1. There were no significant differences between the distal SEH and non-distal SEH groups regarding age, sex, height, weight, BMI, surgical procedure type, hypertension, diabetes mellitus, liver disease, or prior stroke history. Anticoagulant medication use was more frequent in the distal SEH group (66.7% vs. 21.0%), but the difference did not achieve statistical significance (*P* = 0.118).

**Table 1:** Comparison of patient variables according to the presence of postoperative distal SEH.

Characteristic	No distal SEH (*N* = 229)	Distal SEH (*N* = 3)	*P* value
Age	68.43 ± 7.52	63.33 ± 10.07	0.326
Sex (Male, %)	140/229 (61.1%)	2/3 (66.7%)	1.000
Height (cm)	168.84 ± 7.36	169.33 ± 5.13	0.976
Weight (kg)	61.99 ± 9.16	62.00 ± 11.14	0.924
BMI (kg/m^2^)	22.84 ± 2.73	20.83 ± 4.01	0.347
Hemoglobin (g/dL)	12.16 ± 1.24	13.13 ± 1.58	0.340
Hematocrit (%)	41.26 ± 1.45	41.87 ± 0.83	0.410
Platelet (10^9^/L)	263.70 ± 95.28	209.53 ± 99.81	0.345
PT (sec)	11.30 ± 0.92	11.00 ± 1.00	0.550
aPTT (sec)	28.45 ± 1.30	28.47 ± 0.15	0.654
INR	1.01 ± 0.19	1.00 ± 0.10	0.982
Surgery type (Decompression, %)	153/229 (66.8%)	1/3 (33.3%)	0.262
Number of levels exposed (1 level, %)	184/229 (80.3%)	3/3 (100.0%)	1.000
Operative time (min)	111.94 ± 55.58	148.33 ± 72.86	0.374
Estimated blood loss (mL)	143.73 ± 71.72	143.33 ± 40.41	0.612
Drain remove day	2.74 ± 1.13	3.33 ± 1.15	0.319
Hemostatic agent use	33/229 (14.4%)	1/3 (33.3%)	0.380
CSF leakage or dural tear	2/229 (0.9%)	0/3 (0.0%)	1.000
Hypertension	77/229 (33.6%)	0/3 (0.0%)	0.553
Diabetes mellitus	40/229 (17.5%)	0/3 (0.0%)	1.000
Liver disease	16/229 (7.0%)	0/3 (0.0%)	1.000
Stroke history	25/229 (10.9%)	1/3 (33.3%)	0.301
Anticoagulant medication	48/229 (21.0%)	2/3 (66.7%)	0.118
History of PEN	37/229 (16.2%)	3/3 (100.0%)	0.005

Values are mean ± standard deviation or count/denominator (percentage). SEH, spinal epidural hematoma; BMI, body mass index; PT, prothrombin time; aPTT, activated partial thromboplastin time; INR, international normalized ratio; CSF, cerebrospinal fluid; PEN, percutaneous epidural neuroplasty.

Because one cell in the primary distal-SEH 2 × 2 table was zero, the exact odds ratio was not estimable in the conventional finite form and approached infinity. Therefore, the exact Fisher's test *p*-value is reported together with the lower bound of the exact 95% confidence interval. The exact lower bound of the 95% CI is 1.528, indicating that the true odds of developing distal SEH are significantly higher for patients with a history of prior PEN.

### 3.3 Secondary analysis: All SEH cases

In our exploratory univariate analysis of all postoperative SEH cases, a history of prior PEN was more frequent in the SEH group than in the non-SEH group, yielding an exact OR of 15.49 (75.0% vs. 16.2%, Fisher's exact test *P* = 0.017; 95% CI: 1.57–152.99). Baseline characteristics for all postoperative SEH cases are summarized in Table 2. There was no significant association between distal postoperative SEH and surgery type or number of operative levels (Supplementary Tables S1, S2).

**Table 2:** Comparison of patient variables according to the presence of postoperative SEH (secondary analysis).

Characteristic	No SEH (*N* = 228)	SEH (*N* = 4)	*P* value
Age	68.43 ± 7.53	64.25 ± 8.42	0.273
Sex (Male, %)	139/228 (61.0%)	3/4 (75.0%)	1.000
Height (cm)	168.82 ± 7.37	170.25 ± 4.57	0.701
Weight (kg)	62.0 ± 9.2	61.0 ± 9.3	0.829
BMI (kg/m^2^)	22.84 ± 2.73	21.43 ± 3.49	0.310
Hemoglobin (g/dL)	12.16 ± 1.24	12.97 ± 1.33	0.193
Hematocrit (%)	41.26 ± 1.45	41.53 ± 0.96	0.722
Platelet (10^9^/L)	263.98 ± 95.40	207.28 ± 81.62	0.239
PT (sec)	11.30 ± 0.92	11.00 ± 0.82	0.522
aPTT (sec)	28.46 ± 1.29	27.72 ± 1.49	0.259
INR	1.01 ± 0.19	0.95 ± 0.13	0.500
Surgery type (Decompression, %)	152/228 (66.7%)	2/4 (50.0%)	0.604
Number of levels exposed (1 level, %)	183/228 (80.3%)	4/4 (100.0%)	1.000
Operative time (min)	112.17 ± 55.60	126.25 ± 74.09	0.618
Estimated blood loss (mL)	143.86 ± 71.85	136.25 ± 35.91	0.833
Drain remove day	2.75 ± 1.13	3.00 ± 1.15	0.657
Hemostatic agent use	33/228 (14.5%)	1/4 (25.0%)	0.472
CSF leakage or dural tear	2/228 (0.9%)	0/4 (0.0%)	1.000
Hypertension	76/228 (33.3%)	1/4 (25.0%)	1.000
Diabetes mellitus	39/228 (17.1%)	1/4 (25.0%)	0.533
Liver disease	16/228 (7.0%)	0/4 (0.0%)	1.000
Stroke history	25/228 (11.0%)	1/4 (25.0%)	0.380
Anticoagulant medication	48/228 (21.1%)	2/4 (50.0%)	0.204
History of PEN	37/228 (16.2%)	3/4 (75.0%)	0.017

Values are mean ± standard deviation or count/denominator (percentage). SEH, spinal epidural hematoma; BMI, body mass index; PT, prothrombin time; aPTT, activated partial thromboplastin time; INR, international normalized ratio; CSF, cerebrospinal fluid; PEN, percutaneous epidural neuroplasty.

### 3.4 Case descriptions

#### 3.4.1 Case 1

A 54-year-old man presented with chronic low back pain and neurogenic claudication. Preoperative MRI showed lumbar spinal stenosis with spondylolisthesis at L4–5. He underwent transforaminal lumbar interbody fusion (TLIF) with decompressive laminectomy at L4–5 without intraoperative complications.

On postoperative day (POD) 1, he developed acute scrotal pain that was not present preoperatively. Neurological examination showed no significant motor weakness. Because of the atypical symptom pattern and abrupt onset, an urgent postoperative lumbar MRI was obtained on POD 1, revealing an SEH at L5–S1/S2—distal to the operative site—causing significant compression of the thecal sac and sacral nerve roots (Figure 1).

**Figure 1:**
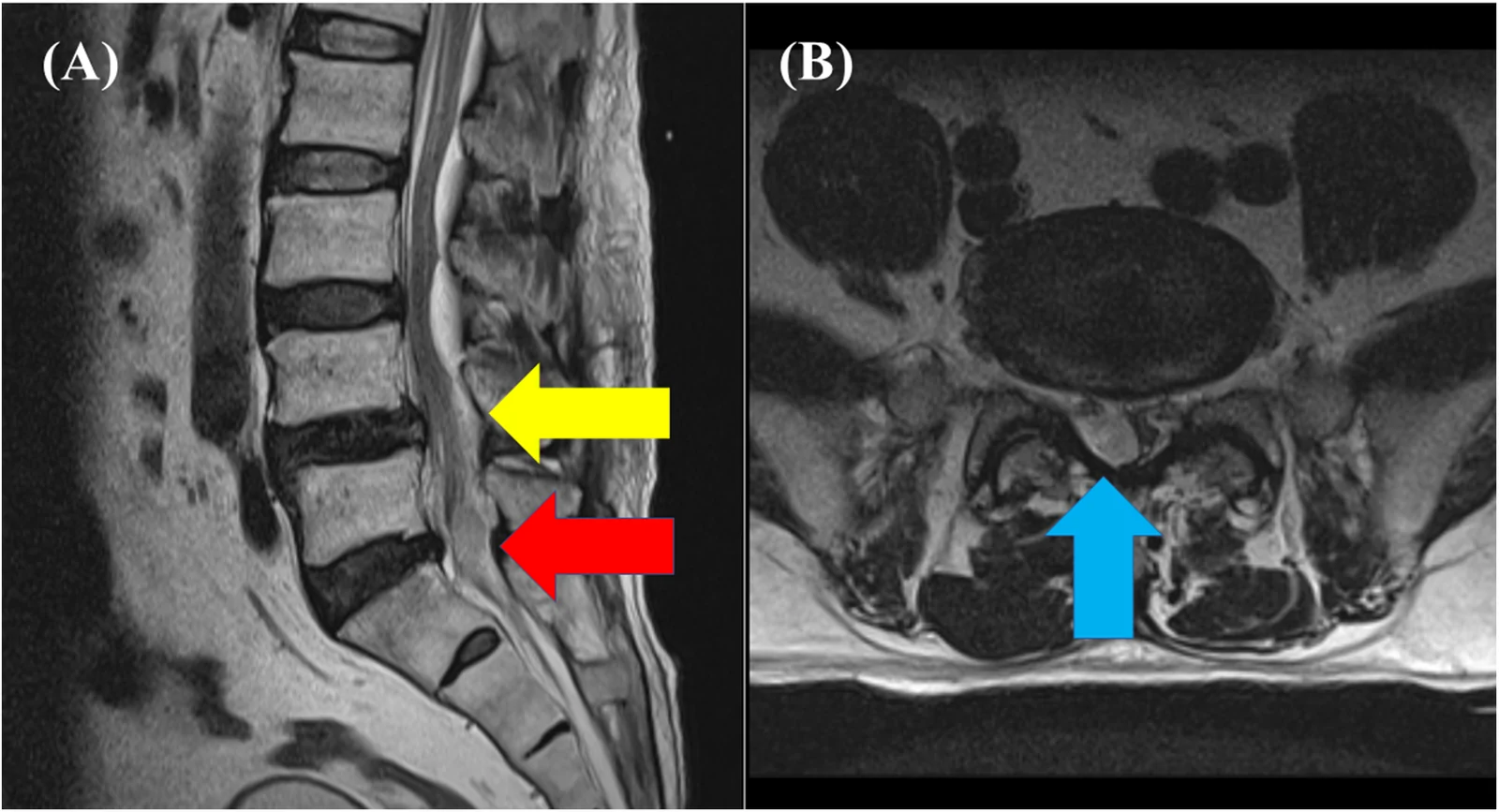
Postoperative lumbar spine magnetic resonance imaging in case 1 showing a distal epidural hematoma. **(A)** T2-weighted sagittal image demonstrating a spinal epidural hematoma at the L5–S1 level, distal to the operative site (yellow arrow), compressing the thecal sac (red arrow). **(B)** Corresponding T2-weighted axial image showing the epidural hematoma at the same level (blue arrow).

He underwent urgent hematoma evacuation through a partial laminectomy at L5 and S1. His scrotal pain improved markedly after surgery and nearly resolved by POD 3, with no additional neurological deficits observed during follow-up.

#### 3.4.2 Case 2

A 62-year-old woman presented with low back pain and left radicular pain. Preoperative imaging showed lumbar spinal stenosis at L3–4. She underwent an uneventful decompressive laminectomy at L3–4 after the failure of conservative treatment.

On POD 1, she developed new lower-extremity weakness accompanied by severe ankle pain, which differed from her preoperative symptoms. Neurological examination confirmed new motor weakness, and an immediate MRI was obtained. The scan showed an SEH at L4–5, completely distal to the decompression site, causing severe compression of the dural sac (Figure 2).

**Figure 2:**
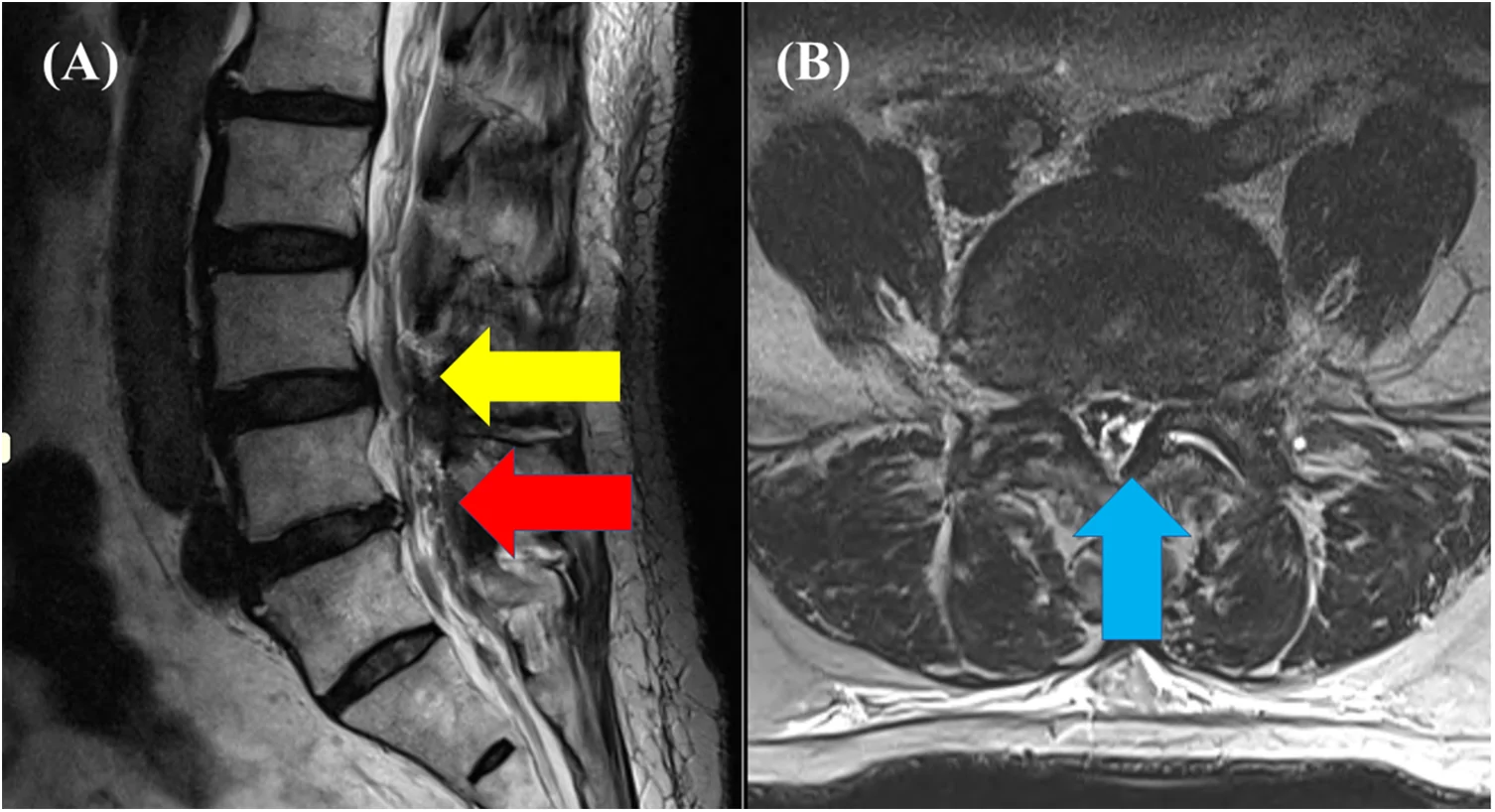
Postoperative lumbar spine magnetic resonance imaging in case 2 showing a distal epidural hematoma. **(A)** T2-weighted sagittal image showing a spinal epidural hematoma at the L4–5 level, distal to the decompression site (yellow arrow), compressing the thecal sac (red arrow). **(B)** T2-weighted axial image at the L4–5 level demonstrating the epidural hematoma with dural sac compression (blue arrow).

She underwent urgent hematoma evacuation via a partial laminectomy at L4. Her motor weakness and radicular pain improved gradually after reoperation, and her neurological status had substantially improved by POD 5.

A comprehensive summary of all four symptomatic SEH cases is provided in Table 3.

**Table 3:** Summary of individual patient findings developing postoperative SEH.

Case	Age/Sex	Surgery	Hematoma level	Prior PEN	Number of prior PEN procedures	Time from last PEN to surgery	Symptom onset	MRI timing	Reoperation timing	Presenting symptom	Outcome
1	54/M	TLIF with decompressive laminectomy, L4-5	L5-S1-S2	Yes	2	1 year	POD #1	16 h after surgery	Urgent	Acute scrotal pain	Near-complete resolution by POD #3
2	62/F	Decompressive laminectomy, L3-4	L4-5	Yes	1	6 months	POD #1	18 h after surgery	Urgent	New lower-extremity weakness, ankle pain	Marked improvement by POD #5
3	74/M	TLIF with decompressive laminectomy, L4-5	L5-S1	Yes	1	2 years	POD #2	50 h after surgery	Urgent	New severe radiating pain	Marked improvement by POD #7
4	67/M	Decompressive laminectomy, L4-5	L4-5	No	-	-	POD #3	66 h after surgery	Urgent	New ankle weakness, radiating pain	Marked improvement by POD #7

SEH, spinal epidural hematoma; PEN, percutaneous epidural neuroplasty; MRI: magnetic resonance imaging, POD, postoperative day; TLIF, transforaminal lumbar interbody fusion.

## 4 Discussion

This study examined postoperative SEH after lumbar surgery for spinal stenosis in a cohort of 232 patients, of whom four developed symptomatic SEH requiring reoperation. Three of these four hematomas occurred at a distal level completely caudal to the operative window. In our secondary exploratory analysis, a history of prior PEN was associated with SEH development (*P* = 0.017, OR: 15.49). However, given the small sample size, wide confidence intervals, and single-center design, this link must be interpreted strictly as an exploratory, hypothesis-generating finding rather than a definitive causal relationship.

The overall incidence of symptomatic SEH in our cohort was 1.72%, which is slightly higher than classic historical series (0.1% to 0.24%) (1, 6, 7). This discrepancy is likely driven by our restrictive inclusion criteria, which selected exclusively for symptomatic patients with severe degenerative spinal stenosis, as well as our small sample layout. Traditional risk factors such as multilevel surgery, coagulopathy, revision surgery, massive blood loss, and anticoagulant exposure were not clearly associated with SEH in this series, but the study was underpowered to evaluate them reliably.

### 4.1 Speculative mechanisms for distal predominance

The biological mechanisms underlying distal level postoperative SEH formation remain speculative, though several physiological dynamics may interact:

#### 4.1.1 Prior pathological substrate

PEN is a nonsurgical epidural intervention performed via the sacral hiatus, advancing a catheter cranially through the ventral epidural space to execute mechanical adhesiolysis and deliver hypertonic saline or corticosteroids (8–10). This process can induce subclinical microvascular alterations, localized fibrosis, or localized venous engorgement along the path of the catheter. Venous channels previously altered or fragile from past catheter interventions may be prone to rupture when exposed to postoperative hemodynamic alterations.

#### 4.1.2 Pressure gradients and dependent pooling

Surgical decompression abruptly alters cerebrospinal fluid and epidural venous pressure dynamics. Postoperative venous bleeding may naturally flow caudally along gravitational gradients, accumulating in the non-decompressed distal space.

#### 4.1.3 Lack of tamponade accommodations

At the index surgical site, the removal of bone and yellow ligament creates a relative compliance volume (“dead space”) that can accommodate minor postoperative bleeding without immediate neural compression. Conversely, the non-decompressed distal levels are tightly confined by intact spinal architecture, meaning even a small volume of migrated or localized blood can trigger rapid neural compression and early symptomatic manifestation.

Alternative explanations cannot be ruled out, including simple statistical variance, occult operative-level hematomas migrating downward, differences in imaging coverage, postoperative patient positioning, drain-related effects, or detection bias (closer monitoring of patients with complex intervention histories). While all three distal cases in this series uniquely shared a history of PEN, this concentration may reflect these underlying selection, detection, or confounding dynamics rather than a true independent causal relationship.

### 4.2 Limitations

This study has several limitations. First, it was retrospective and based on a small sample with only four events, including only three distal SEH cases. Second, granular historical details regarding prior PEN procedures, such as the target level, catheter tip level, injectates, volume, precise catheter path, and disease burden, were not uniformly retrievable from the records. Third, certain perioperative parameters, such as transient postoperative blood pressure spikes, intraoperative vasopressor requirements, and exact mobilization timeframes, could not be uniformly extracted from the historical charts, leaving residual confounding variables unexamined. Furthermore, postoperative imaging was ordered at the surgeon's discretion based on clinical deterioration rather than by routine protocol, so asymptomatic or mildly symptomatic distal collections may have been missed.

In addition, the primary distal SEH analysis included a zero cell, which inflates apparent association and widens the confidence interval, further limiting interpretability. Finally, because the analysis was unadjusted, residual confounding by patient and surgical factors is likely. For these reasons, the findings should be regarded as hypothesis-generating only.

Despite these limitations, the study raises a clinically relevant point. In postoperative patients with a history of PEN who develop atypical pain or new neurological symptoms, MRI should not necessarily be limited to the operated level. Extending imaging caudally may help avoid missed distal SEH in selected cases.

## 5 Conclusion

In conclusion, this small case series shows an exploratory association between prior PEN and symptomatic distal postoperative SEH requiring reoperation. Because of the limited number of events, the zero-cell structure of the primary 2 × 2 table, and the wide confidence intervals, causal inference is not possible. In postoperative patients with a history of prior caudal epidural catheter interventions who develop atypical pain or new neurological symptoms, diagnostic imaging should be considered beyond the index surgical margins to avoid missing a distal hematoma.

## Data Availability

The original contributions presented in the study are included in the article/Supplementary Material, further inquiries can be directed to the corresponding author/s.

## References

[1] Uribe J Moza K Jimenez O Green B Levi ADO. Delayed postoperative spinal epidural hematomas. Spine J. (2003) 3:125–9. 10.1016/s1529-9430(02)00535-1 14589226

[2] Griessenauer CJ Raborn J Foreman P Shoja MM Loukas M Tubbs RS. Venous drainage of the spine and spinal cord: a comprehensive review of its history, embryology, anatomy, physiology, and pathology. Clin Anat. (2015) 28:75–87. 10.1002/ca.22354 24677178

[3] Carpenter K Decater T Iwanaga J Maulucci CM Bui CJ Dumont AS. Revisiting the vertebral venous plexus-A comprehensive review of the literature. World Neurosurg. (2021) 145:381–95. 10.1016/j.wneu.2020.10.004 33049379

[4] Kou J Fischgrund J Biddinger A Herkowitz H. Risk factors for spinal epidural hematoma after spinal surgery. Spine. (2002) 27:1670–3. 10.1097/00007632-200208010-00016 12163731

[5] Vasankari V Oulasvirta E Krishnan A Resendiz-Nieves J Marjamaa J Antinheimo J. Risk factors for postoperative epidural hematoma after microsurgical decompression of lumbar spinal stenosis. Brain Spine. (2026) 6:106020. 10.1016/j.bas.2026.106020 41952955 PMC13054050

[6] Scavarda D Peruzzi P Bazin A Scherpereel B Gomis P Graftieaux JP. Postoperative spinal extradural hematomas. 14 cases. Neurochirurgie. (1997) 43:220–7. 9686224

[7] Lawton MT Porter RW Heiserman JE Jacobowitz R Sonntag VK Dickman CA. Surgical management of spinal epidural hematoma: relationship between surgical timing and neurological outcome. J Neurosurg. (1995) 83:1–7. 10.3171/jns.1995.83.1.0001 7782824

[8] Manchikanti L Rivera JJ Pampati V Damron KS McManus CD Brandon DE. One day lumbar epidural adhesiolysis and hypertonic saline neurolysis in treatment of chronic low back pain: a randomized, double-blind trial. Pain Physician. (2004) 7:177–86. 10.36076/ppj.2004/7/177 16868590

[9] Manchikanti L Pampati V Fellows B Rivera J Beyer CD Damron KS. Role of one day epidural adhesiolysis in management of chronic low back pain: a randomized clinical trial. Pain Physician. (2001) 4:153–66. 10.36076/ppj.2001/4/153 16902688

[10] Veihelmann A Devens C Trouillier H Birkenmaier C Gerdesmeyer L Refior HJ. Epidural neuroplasty versus physiotherapy to relieve pain in patients with sciatica: a prospective randomized blinded clinical trial. J Orthop Sci. (2006) 11:365–9. 10.1007/s00776-006-1032-y 16897200

