# Supplementary material for: Association between prior percutaneous epidural neuroplasty and distal postoperative spinal epidural hematoma after lumbar surgery: case series and exploratory analysis
Source: Front Surg. 2026 Sep 24;13:1863930. doi: 10.3389/fsurg.2026.1863930 (PMC13646040; doi:10.3389/fsurg.2026.1863930)
Supplement: Supplementary file 1 [file Table1.docx]

| **Supplementary Table 1. Stratification of clinical parameters according to surgery type** | | | |
| --- | --- | --- | --- |
|  | Decompression (N = 154) | Fusion (N = 78) | P value |
| Age | 68.99 ± 7.24 | 67.12 ± 8.02 | 0.084 |
| Sex (Male, %) | 96/154 (62.3%) | 46/78 (59.0%) | 0.67 |
| Distal SEH | 1/154 (0.6%) | 2/78 (2.6%) | 0.262 |
| History of PEN | 22/154 (14.3%) | 18/78 (23.1%) | 0.101 |
| Operative time (min) | 77.79 ± 22.67 | 180.77 ± 34.43 | <0.001 |
| Estimated blood loss (ml) | 135.91 ± 72.73 | 159.17 ± 67.41 | 0.016 |
| Drain remove day | 2.11 ± 0.49 | 4.01 ± 0.96 | <0.001 |
| Hemostatic agent use | 17/154 (11.0%) | 17/78 (21.8%) | 0.032 |
| CSF leakage or dural tear | 0/154 (0.0%) | 2/78 (2.6%) | 0.112 |
| Values are mean ± standard deviation or count/denominator (percentage). SEH: spinal epidural hematoma, PEN: percutaneous epidural neuroplasty, CSF: cerebrospinal fluid | | | |

| **Supplementary Table 2. Stratification of clinical parameters according to the number of operative levels** | | | |
| --- | --- | --- | --- |
|  | 1 level (N = 187) | 2 levels (N = 45) | P value |
| Age | 68.43 ± 7.54 | 68.07 ± 7.66 | 0.774 |
| Sex (Male, %) | 115/187 (61.5%) | 27/45 (60.0%) | 0.866 |
| Distal SEH | 3/187 (1.6%) | 0/45 (0.0%) | 1.000 |
| History of PEN | 31/187 (16.6%) | 9/45 (20.0%) | 0.66 |
| Operative time (min) | 104.60 ± 53.23 | 144.89 ± 54.98 | <0.001 |
| Estimated blood loss (ml) | 114.36 ± 37.07 | 265.78 ± 44.94 | <0.001 |
| Drain remove day | 2.85 ± 1.19 | 2.33 ± 0.71 | <0.001 |
| Hemostatic agent use | 27/187 (14.4%) | 7/45 (15.6%) | 0.817 |
| CSF leakage or dural tear | 1/187 (0.5%) | 1/45 (2.2%) | 0.351 |
| Values are mean ± standard deviation or count/denominator (percentage). SEH: spinal epidural hematoma, PEN: percutaneous epidural neuroplasty, CSF: cerebrospinal fluid | | | |
